# Inflammation and Macular Oedema after Pars Plana Vitrectomy

**DOI:** 10.1155/2013/971758

**Published:** 2013-10-30

**Authors:** Vito Romano, Martina Angi, Fabrizio Scotti, Renata del Grosso, Davide Romano, Francesco Semeraro, Paolo Vinciguerra, Ciro Costagliola, Mario R. Romano

**Affiliations:** ^1^Second University of Naples, Via Pansini 5, 80100 Napoli, Italy; ^2^Department of Molecular and Clinical Cancer Medicine, University of Liverpool, Liverpool, UK; ^3^Department of Ophthalmology, Istituto Clinico Humanitas, Rozzano, Milan, Italy; ^4^Ophthalmology Unit, San Sebastiano Hospital, Caserta, Italy; ^5^Eye Clinic, Department of Neurological Sciences and Vision, University of Brescia, Brescia, Italy; ^6^Eye Clinic, Department of Health Sciences, University of Molise, Campobasso, Italy

## Abstract

Cystoid macular oedema (CMO) is a major cause of reduced vision following intraocular surgery. Although the aetiology of CMO is not completely clarified, intraocular inflammation is known to play a major role in its development. The macula may develop cytotoxic oedema when the primary lesion and fluid accumulation occur in the parenchymatous cells (intracellular oedema) or vasogenic oedema when the primary defect occurs in the blood-retinal barrier and leads to extracellular fluid accumulation (extracellular oedema). We report on the mechanisms of CMO formation after pars plana vitrectomy and associated surgical procedures and discuss possible therapeutic approaches.

## 1. Introduction

Macular oedema results from serous exudation of incompetent intraretinal capillaries localized between the retina's outer (plexiform) and inner (nuclear) layers, as well as from swelling in retinal Müller cells. Cystoid macular oedema (CMO) is a localized expansion of the extracellular, and sometimes intracellular, space in the macular area of the retina and has a characteristic radially orientated cystic pattern with perifoveal cyst-like spaces [1]. The empty space may result in lamellar holes or full-thickness oedema, which consequently damages the outer retinal layers resulting in permanent central vision impairment [1–3]. CMO can arise in cases of central or branch retinal vein occlusions, diabetic retinopathy, and retinal traction disorders due to blood-retinal barrier (BRB) alterations [4].

BRB alterations are the result of cytotoxic insult that is secondary to intraocular inflammation. The same mechanism appears to be responsible for iatrogenic damage after cataract extraction and other kinds of intraocular surgeries, such as vitreoretinal surgery [2]. The BRB is located on two levels: the chorioepithelial interface and the retinal vessels, forming the outer and inner BRB, respectively. The retinal pigment epithelium of the outer BRB is comprised of cells linked by tight junctions, adherent junctions, and desmosomes. The endothelial membrane of the retinal vessels of the inner BRB is comprised of cells linked by tight junctions. Together, the retinal pigment epithelium and the endothelial membrane form the BRB's main structures. Under physiological conditions, the BRB separates blood from the surrounding retinal tissue and maintains environmental stability for ocular neurons and photoreceptors by controlling the movement of proteins and cells from the blood into these tissues [5]. Additionally, every neuron and glial cell has a membrane transport system that balances ion and water movement in and out of the cell [5]. 

Under pathological conditions, the retina may develop cytotoxic oedema, where the primary lesion and fluid accumulation occur in the parenchymatous cells (intracellular oedema), or vasogenic oedema, where the primary defect occurs in the BRB and leads to extracellular fluid accumulation (extracellular oedema) [6]. The vasogenic damage that occurs in vasogenic oedema is governed by inflammatory cells, such as macrophages, neutrophils, and several other inflammatory mediators. These mediators include angiotensin II, vascular endothelial growth factor (VEGF), prostaglandins, cytokines, chemokines, matrix metalloproteinases, interleukins, P-selectin, E-selectin, VCAM-1, and ICAM-1 [7, 8]. Typically, although some conditions primarily cause extracellular oedema or intracellular oedema, a hybrid of both types of oedemas occurs simultaneously.

In this paper, we report on the mechanisms of CMO formation after pars plana vitrectomy and associated surgeries and discuss possible therapeutic approaches.

## 2. Cystoid Macular Oedema after Pars Plana Vitrectomy

The overall incidence of CMO after pars plana vitrectomy (PPV) is not easily determined, as it is often related to previous conditions, such as central or branch retinal vein occlusions, diabetic retinopathy, and retinal traction disorders. The most accurate data come from patients undergoing PPV for vitreous floaters, where any postoperative CMO is clearly linked to this surgical procedure. The work carried out by de Nie et al. on this topic showed that CMO after PPV occurred in 5.5% of cases. All patients were successfully treated with medical treatment, except two cases that needed a second surgery [9]. Other studies with the same inclusion criteria did not record any case of CMO after PPV [10–12]. These data show that the technical developments over the past years have made vitrectomy a mini-invasive type of surgery, improving the risk/benefit equation.

## 3. Cystoid Macular Oedema after Pars Plana Vitrectomy with Internal Limiting Membrane Peeling

Optical coherence tomography (OCT) and histological findings provide detailed retinal microstructure imaging. They help in delineating any inflammatory damage occurring after PPV, the role played by the internal limiting membrane (ILM), and any benefits of ILM removal during surgery. The interstitial pathway from the vitreous cavity to the subretinal space is formed by an external and an internal limiting membranes. The junctions between the photoreceptors and the Müller cells of the external limiting membrane (ELM) are not sealed and, consequently, can only partially limit the movement of large molecules. However, the ILM has no significant influence on water movement. The balance between static and dynamic vitreous tractional forces determines whether CMO forms a macular hole or becomes a chronic tractional CMO [13].

ILM peeling may have beneficial effects on CMO because it removes tangential traction, increases retinal oxygenation, reduces VEGF production, and allows intraretinal fluid from the macula to reach the vitreous cavity [14]. Studies have shown that the Müller cells immediately swell (intracellular oedema) after PPV with ILM peeling and that this swelling persists. However, Kado et al. showed that the period of macular oedema (extracellular oedema) could be shortened by reducing the centripetal traction transmitted to the Müller cells by vitreous fibres inserted into the macula [15]. Additionally, ILM removal may also help preventing postoperative complications [16, 17]. Spaide recently observed an inner retinal dimple along the path of the nerve fiber layer in 52% of the eyes treated with ILM peeling [18]. The Müller cell footplates run over the inner surface of the nerve fiber layer, having the ILM as a basement membrane. The patients developed a radiating pattern of darker spots within a thin superficial grayish lamina. This pattern has been called dissociated optic nerve fiber layer (DONFL) appearance and it seems to be related to the impact of Müller cell footplates avulsion [18]. DONFL has been also described by Tadayoni et al. after epiretinal membrane (ERM) removal [19]. The authors described slightly darker arcuate striae in the direction of the optic nerve fibers. This feature had no functional effect on postoperative functional prognosis [18, 19].

PPV with ILM peeling in retinal vein occlusions removes traction and reduces VEGF and IL-6 production, two factors responsible for inducing vascular permeability [15, 20, 21]. Mandelcorn et al. [22, 23] have hypothesized that PPV-ILM peeling decompresses retinal blood vessels, thereby facilitating the release of extracellular fluid and blood into the vitreous cavity, where it can be more easily removed. Other authors have also highlighted the lack of ERM formation and CMO recurrence following this surgery [24]. Raszewska-Steglinska et al. reported that 68% of patients in their series had improved visual acuity after PPV-ILM peeling and that the best results were obtained in patients treated within 1 month of CMO onset [16].

ILM peeling has also been associated with PPV for the treatment of retinal detachment (RD) with proliferative vitreoretinopathy (PVR), in the hope of reduceing postoperative CMO. A retrospective study of 90 eyes demonstrated a reduction in CMO in some patients; however, PPV-ILM peeling was still not enough to eliminate this complication in 47% of cases [25]. Better results were shown by Schocket et al., who reported CMO in only 12% of eyes treated for RD [26], and by Kiss et al. (17%) [27]. The RD duration, the numbers of surgeries, and the mechanical activities related to ILM peeling were important in both these situations [25]. Chang et al. further confirmed that apoptosis and macular oedema begin a few hours after RD and that apoptosis and oedema severity only increase by time to significantly influence visual acuity [28, 29].

In contrast to the above, the postoperative retinal thickness and visual acuity of diabetic patients after PPV-ILM peeling were not significantly better than those of the ILM-preserved group in two Japanese studies [30, 31]. In these patients, however, attention must also be paid to preexisting ocular conditions (i.e., diabetic retinopathy, uveitis, and/or a preexisting ERM) and systemic risk factors (e.g., renal failure and hypertension) because these can influence the prognosis of diabetic CMO [32]. These conditions can lead to vascular instability, mostly due to endothelial cell damage by advanced glycosylation end-products, which predispose the BRB to breaking down.

An immunohistochemical study of ILMs peeled during vitrectomy for various aetiologies found strong adhesions between ILM cells and, consequently, that ILM peeling increases the risk of removing inner retinal structures [33]. ERM formation involves epiretinal glial proliferation and induces significant intraretinal changes [34]. This has been associated with increased expression of the intermediate filament protein GFAP in both Müller cells and astrocytes [35]. GFAP forms bridges between the cytoskeleton, epiretinal receptors, and the extracellular matrix [36]. Thus, the GFAP within Müller cells may alter adhesion between these cells and the ILM. Consequently, removing the ILM may damage Müller cells and transmit a focal force towards the inner retina that results in the avulsion of some retinal cells and the loss of competent retinal structure. This may increase the propensity for developing CMO if additional intraocular inflammation occurs [33, 37]. 

## 4. Cystoid Macular Oedema after Pars Plana Vitrectomy and Cataract Surgery

Patients who have already undergone PPV with epiretinal peeling have a higher incidence of CMO after a second intraocular surgery [38]. A prospective, nonrandomized, controlled clinical study found that 26% of eyes developed CMO after successful cataract surgery when eyes had been previously treated with PPV and ERM and ILM peeling [38]. In contrast, no cases of CMO were observed in the control group. The problem is mainly related not to the combination of surgeries but to the lack of vitreous and of a competent retinal structure. Therefore, cataract surgery should be avoided after vitrectomy and, instead, be planned before or at the same time of PPV [38, 39].

Even though combined vitrectomy presents its advantages in regard to CMO formation, it has several disadvantages as well. The main disadvantages are increased postoperative inflammation and the complications related to such inflammation. This holds particularly true in diabetic patients, where a higher incidence of postoperative complications (such as synechia formation and fibrinous uveitis) has been reported following combined phaco/vitrectomy; especially if the retinopathy is very active, a large amount of intraoperative laser is needed or tamponade is used [40–47]. In such patients subconjunctival and topical steroids can be used at the end of the surgery to lessen the incidence of these complications.

Jiramongkolchai et al. retrospectively evaluated the incidence of macular oedema and cataract formation after PPV in diabetic patients who required cataract surgery. Macular oedema incidence was 6% six months after PPV and 30% six months after cataract surgery in the same patients. This suggests that factors independent of the vitreous, such as inflammation, are mainly involved in the pathogenesis of macular oedema after cataract surgery in diabetics [48]. Additionally, according to Bhatnagar et al., patients who have already undergone surgery for macular holes have an increased risk for macular hole recurrence after cataract surgery [49]. This is most likely due to ILM peeling causing a loss of retinal structure and greater responsiveness to inflammatory stimuli. Consequently, CMO recurs and the macular hole reopens. In other studies, no association was found between cataract extraction and macular hole reopening [50–52]. However, this situation is unclear, because several differences exist between the design and inclusion criteria of these studies that may explain the discrepant results.

## 5. Cystoid Macular Oedema after Silicone Oil Removal

The use of silicon oil (SiO) as a long-term intraocular tamponade may lead to macular changes such as CMO. A comparative analysis of macular microstructures before and after SiO removal reported that microstructural changes were associated with the duration of SiO tamponade and that most of the microstructural changes were reversed upon SiO removal. Under SiO tamponade, the OCT identified CMO in 19.6% of cases. In most cases, however, visual acuity was significantly improved after SiO removal in correlation with the decrease of CMO [53]. In one retrospective interventional case series, complicated RD with PVR macular changes was observed in 87% of patients following SiO removal, and 18% of those had CMO that required additional treatment [27]. Cox et al. also showed that the CMO is not related to epiretinal traction since ERM formation was not statistically related to the type of tamponade (SiO versus gas) [54].

SiO impurities, such as the oil's low molecular weight components (LMWC) and residual catalysts, are thought to cause the ocular inflammation. Using gas chromatography, Nakamura et al. analysed SiO up to two years after injection and found evidence of decreased LMWC concentrations. LMWC likely diffused from the oil into the ocular tissues, resulting in chronic ocular toxicity [55]. Furthermore, histopathological analysis of an ERM that developed after intraoperative use of perfluorocarbon liquids identified an inflammatory reaction with foreign body response to intraocular tamponade [56]. 

## 6. Cystoid Macular Oedema after Pars Plana Vitrectomy for Retained Lens Fragments

Clinical CMO occurs in fewer than 2% of eyes after an uneventful cataract surgery and rarely becomes chronic [57, 58]. Conversely, clinical CMO is reported in up to 28% of eyes after PPV for retained lens fragments and becomes chronic in about 20% of these eyes [59]. If residual fragments were not removed from the eye, the incidence of CMO would likely be even higher [60]. Moreover, after vitreous removal, the eye behaves like a single compartment. Therefore, in vitrectomized eyes, inflammatory mediators can more easily diffuse from the iris and anterior chamber to the macula, causing CMO [61]. Furthermore, the lens epithelial cells (LECs) are responsible for synthesis of prostaglandins and cytokines such as PGE2, IL-1, and TGF-beta [62].

Posterior dislocation of nuclear lens fragments is associated with a worse visual outcome than that of nonnuclear fragments. This is likely due to direct mechanical damage to the retina, a stronger inflammatory response, or a more traumatic vitrectomy procedure [63]. A retrospective study of 91 patients who had PPV for retained lens fragments observed that CMO developed in only 8% of patients with a sulcus-fixated posterior chamber intraocular lens. In contrast, CMO developed in 46% of patients with aphakia or an anterior chamber intraocular lens (IOL) [64]. In these cases, long-term anti-inflammatory therapy should be considered because of the high rate of CMO recurrence.

The timing of surgical retained lens fragment removal remains a multifactorial decision involving surgeon and patient preferences, situational logistics, and clinical judgment. A systematic review and meta-analysis of retrospective interventional cases found evidence that postoperative outcomes, such as visual acuity, RD, increased intraocular pressure, and intraocular infection/inflammation, are better with early PPV [65]. However a retrospective study on 569 eyes found similar visual acuity outcomes and complication rates in patients undergoing same-day or a later PPV [66].

## 7. Cystoid Macular Oedema in the Presence of Epiretinal Traction

Some reports emphasize the role of mechanical factors in clinical CMO. These factors include tractional forces on the macula (i.e., ERM or vitreomacular traction) that pull on the retinal surface resulting in vascular damage and in the release of mediators which lead to the breakdown of the BRB. Vitreomacular traction syndrome (VMT) can, therefore, cause both tractional and exudative CMO [67, 68]. Prognosis and treatment options depend on the size and configuration of the residual vitreomacular adhesion and on the consequential anatomical macular changes [69]. This type of CMO can be easily confused with postoperative, uveitic, or retinal vascular CMO [70]. Important clinical clues of tractional etiology may include metamorphopsia, subtle asymmetry of the cystoid foveal thickening, and the absence of leakage via fluorescein angiography. Surgical intervention for this CMO appears to benefit the majority of patients with significant associated visual loss. 

## 8. Medical Treatment for Cystoid Macular Oedema

The rationale for pharmacological CMO treatment after vitreoretinal surgery is based on understanding the aetiology and inhibition of these pathophysiological mechanisms. The main factor triggering CMO is the release of inflammatory mediators; vitreous traction does not always play a role in the CMO pathogenesis. Other possible mechanisms include photoretinal stress and pathologic evidence of Müller cell damage. However, more research is needed to better understand the cause of CMO and its pathophysiology [33]. 

CMO treatment aims to reduce the release of inflammatory mediators which results from the breakdown of the BRB. These mediators generate vasogenic damage such as vasodilation, increased capillary permeability, leukocyte migration, and finally CMO [71].

Nonsteroidal anti-inflammatory drugs (NSAIDs) inhibit cyclooxygenase 1 and 2 and, therefore, prostaglandin production. Thus, NSAIDs modulate fluid movement coupled with chloride movement. Cyclooxygenase inhibitors (e.g., indomethacin and other NSAIDs) reduce the incidence of angiographic CMO [72]. The ability of topical NSAIDs to penetrate ocular tissues, including retinal tissue, is an important factor for treating and preventing CMO. NSAID use has been beneficial for chronic postoperative macular oedema. Flach found that a topical NSAID (0.5% ketorolac tromethamine) was effective and that treatment duration of three months provided a more persistent benefit than one or two months [73, 74]. Baklayan et al. showed that Xibrom, a highly lipophilic ophthalmic solution of 0.09% bromfenac, rapidly penetrates ocular tissues [75]. This resulted in both rapid and sustained detectable drug levels in all relevant ocular tissues, including the retina, for over 24 h following a single topical administration. The efficacy of topical NSAIDs in treating CMO has been reviewed in great detail elsewhere. The general consensus is that, despite the paucity of well-designed studies, NSAID treatment is beneficial by reducing macular oedema and possibly improving vision, at least in the short term [72].

Corticosteroids are also well known for their effects on inflammation and cellular proliferation. Corticosteroids block phospholipase A, which acts upstream the arachidonic acid cascade. Consequently, they also block prostaglandin and leukotriene production, downregulate VEGF, and decrease occludin phosphorylation, thereby increasing the tightness of the BRB [76, 77]. Systemic steroid treatment does not seem to significantly improve the anatomic and functional outcomes of CMO [78]. However, periocular application or intravitreal injections appear to be effective for CMO management [79–82]. A prospective randomized, controlled trial of 315 patients with persistent macular oedema due to uveitis or Irvine-Gass syndrome showed that 700 mg of intravitreous dexamethasone over 90 days was well tolerated and resulted in statistically significant improvements in visual acuity and vascular leakage compared to a 350 mg dose [83]. 

Experimental studies have shown that the vitreous half-life of different drugs after intravitreal injection decreases after PPV [84]. The corticosteroid triamcinolone acetonide has been used during vitrectomy to prevent postoperative inflammatory complications [85]. Intravitreal triamcinolone acetonide was more rapidly cleared in vitrectomized patients, though. Schinder et al. suggested that triamcinolone acetonide in the empty vitreous cavity can circulate more easily and faster than that in the normally viscous vitreous [86]. The vitreous is made of highly viscous, gel-like materials, and intravitreal corticosteroids are condensed into a small space. Consequently, highly viscous vitreous likely has a very slow gel circulation. Therefore, the widespread distribution and increased circulation of triamcinolone acetonide in an empty vitreous cavity may be responsible for its rapid clearance. 

In contrast, Chang-Lin et al. reported that the vitreoretinal pharmacokinetic profiles of a dexamethasone intravitreal implant were similar between nonvitrectomized and vitrectomized eyes [87]. In both groups, the decrease in central retinal thickness was usually accompanied by improved visual acuity, and no systemic side effects were observed. However, ocular side effects developed in 70.6% of patients, including increased intraocular pressure (47.1%), transient hypotony (11.8%), displacement of the implant into the anterior chamber in aphakic eyes (5.9%), and RD (5.9%) [87]. 

Topical betaxolol is a *β*1-selective adrenoceptor antagonist with ocular hypotensive and retinal neuroprotective effects. It is also a vasodilator that acts by blocking Ca^2+^ channels. Consequently, betaxolol may play a role in relaxing retinal microarteries, which would improve ocular circulation, resolve macular oedema, and restore retinal function. A randomized clinical trial noted that topical betaxolol appeared to have a favourable effect for eyes with macular oedema [88]. 

Carbonic anhydrase inhibitors are widely used for modulating the polarized distribution of carbonic anhydrase in retinal pigment epithelium. This occurs via extracellular pH gradients and stimulates fluid resorption from the retina to the choroid. Anti-VEGF agents can also restore occludin proteins in the BRB and reduce protein kinase C activation. 

Heier et al. suggested that a combination of topical ketorolac and steroids appeared to offer benefits over monotherapy for acute CMO [89]. Additionally, three additional small studies, which could not be directly compared, have also indicated that using topical anti-inflammatory drugs in combination with topical steroids has therapeutic benefits [3].

Evidence for treating acute CMO remains insufficient for recommending any practices as an adequate solution.

## 9. Prevention of Cystoid Macular Oedema

CMO can lead to permanent structural damage of the outer nuclear layers therefore causing irreversible visual loss. Minimally traumatic and fast vitreoretinal surgery is the primary means of preventing CMO. 

Attention must be paid to preexisting systemic conditions such as diabetes and cerebrovascular and cardiovascular diseases as well as to preexisting ocular conditions [90]. In these cases, using NSAIDs as a prevention strategy may be effective for preventing CMO [73, 74, 91, 92]. Several topical NSAIDs are commercially available for ophthalmic use. Heier et al. measured vitreous drug levels in patients who received either 0.4% ketorolac, 0.09% bromfenac, or 0.1% nepafenac for three days before vitrectomy surgery. All three NSAIDs were able to penetrate the vitreous cavity. Additionally, they found that ketorolac might have a clinical impact on managing prostaglandin-mediated diseases, including CMO [93]. Preoperative NSAID use can also stabilize pupillary dilation during intraocular surgery and reduce postoperative inflammation, pain, and the occurrence of CMO [39, 72]. 

In conclusion, preventing intraocular inflammation appears to be more successful than curing CMO. Prevention should be initiated 6 weeks in advance for uncomplicated surgery or 3 months for complicated surgery and in cases where risk factors are a concern. 
